# Retinoblastoma in Ethiopian Children: Imaging Findings and Staging

**DOI:** 10.4314/ejhs.v34i1.7S

**Published:** 2024-10

**Authors:** Abebe Mekonnen Woldeyohannes, Biruk Abebe Wondimu, Daniel Hailu Kefenie, Tesfaye Kebede Legesse, Semira Abrar Issa

**Affiliations:** 1 Radiologists, Addis Ababa University, College of Health Sciences, Medical Faculty, Department of Radiology, Addis Ababa, Ethiopia; 2 Pediatrician, Addis Ababa University, College of Health Sciences, Medical Faculty, Department of Pediatrics, Addis Ababa, Ethiopia

**Keywords:** Retinoblastoma, Neuroimaging, MRI, CT scan, Ethiopia, Africa

## Abstract

**Background:**

Retinoblastoma is the most prevalent intraocular retinal malignancy in children worldwide. Accurate staging is critical for treatment planning and relies heavily on radiologic imaging and clinical findings. This study aims to evaluate imaging patterns and staging of retinoblastoma in children at Tikur Anbessa Specialized Hospital (TASH).

**Materials and Methods:**

This cross-sectional study analyzed retrospective data from TASH between September 2018 and September 2021. It focused on patients diagnosed with retinoblastoma who underwent Computed Tomography (CT) scans or Magnetic Resonant Imaging (MRI) and had pathology results available. Two radiologists, each with over 10 years of experience, independently reviewed the scans. Supplementary data were gathered from the pediatric oncology unit registry using structured questionnaires. The International Retinoblastoma Staging System (IRSS) was used to stage extraocular disease based on cross-sectional imaging and the International Intraocular Retinoblastoma Classification (IIRC) for intraocular disease classification.

**Results:**

Eighty-three patients were included, with 42 (50.6%) males and 41 (49.4%) females. The mean age at presentation was 3.4 ± 2 years. The most common clinical symptoms were proptosis (42 patients, 50.6%) and leukocoria (37 patients, 44.6%). Clinical staging revealed 63 (75.9%) patients in Group E, 19 (22.9%) in Group D, and 1 (1.2%) in Group C according to IIRC. Stage IV disease was predominant, with 33 (39.2%) in Stage IVA and 18 (21.4%) in Stage IVB according to IRSS.

**Conclusion:**

Most patients presented with advanced retinoblastoma, particularly Stage IV. This underscores the need for community awareness of early signs and symptoms of retinoblastoma, promoting timely medical consultation.

## Introduction

Retinoblastoma is a malignant neoplasm that originates from the retina. It stands as the most prevalent intraocular malignancy in infants and children worldwide, with the majority of cases manifesting before the age of 2(1) There is an estimated 9000 new retinoblastoma cases annually (2).

Retinoblastoma arises due to mutations in the RB1 gene, recognized as a tumor suppressor gene. The loss of one allele of RB1 makes an individual predisposed to retinoblastoma. While RB1 loss signifies the potential for a susceptible retinal cell to turn malignant, it typically results in retinoma, a benign precursor to retinoblastoma. The transition from benign retinoma to malignant retinoblastoma is not identified. Some researchers speculate that it could be attributed to the accumulation of genomic instability (3, 4)

Currently, no specific regions or populations with a predisposition to retinoblastoma have been identified. The disease burden is most pronounced in regions with high birth rates, particularly in Africa and Asia. In these areas, mortality rates are also notably elevated, with over 40-70% of children diagnosed with retinoblastoma succumbing to the disease. This starkly contrasts with the situation in Europe and North America, where only 3-5% of children with the condition face a similar outcome (5). The high mortality in developing countries is primarily linked to delay in diagnosis, typically exceeding 6 months (6).

The primary clinical indicator of retinoblastoma is leukocoria, characterized by a white pupillary reflex. Other common manifestations include strabismus and diminished vision. Advanced cases may exhibit alterations in the globe eye and corneal enlargement, and, in the most severe instances, exophthalmos (2, 6).

The morbidity and mortality associated with retinoblastoma are linked to the disease stage at the time of presentation. Children who present early exhibit a survival rate exceeding 95%, whereas those who present late face a significantly worse prognosis, with mortality reaching up to 70% (70). A study conducted in Ethiopia revealed that the most common presenting symptom is exophthalmos, indicative of late-stage presentation and, consequently, a poorer outcome for the affected children (8).

Effective tumor staging in orbital pathologies is achieved through cross-sectional imaging techniques like computed tomography (CT) and magnetic resonance imaging (MRI). Cross-sectional imaging also provides vital information on the characteristics and extent of the lesion, complementing the clinical ophthalmologic evaluation which is vital for reaching a conclusive diagnosis. Advancements in these modalities facilitate the integration of CT and MRI images into radiation planning systems, enhancing overall diagnostic precision and treatment planning (9).

Over the years, various staging schemes have been employed for retinoblastoma. The initial classifications were introduced by Rees and Ellsworth in 1980, followed by the adoption of the International Intraocular Retinoblastoma Classification (IIRC) in 2003 (10). Currently, it is recommended to utilize the IIRC classification for assessing intraocular disease at the time of diagnosis in each eye(6). Simultaneously, the International Retinoblastoma Staging System (IRSS) classification system is used as the main classification scheme for evaluating the extent of extraocular disease which combines surgical treatment, histopathologic outcome, and clinical findings(10).

The management of retinoblastoma is intricate and is most effectively carried out through a multidisciplinary approach involving pediatric oncologists, ophthalmologists, pediatric nurses, radiologists, pathologists, and social workers (6).

Despite the numerous studies conducted in Western countries on the characteristics and staging of retinoblastoma, there is a paucity of literature examining the patterns of retinoblastoma in sub-Saharan Africa (11). Additionally, there is a notable absence of studies detailing the imaging patterns of retinoblastoma in patients within our specific healthcare setup. We aimed to address this gap by investigating both the imaging pattern and stage at diagnosis of retinoblastoma in our local context.

## Methods and Materials

**Study area and design**: This research was conducted at the Pediatric Oncology Clinic of Tikur Anbessa Specialized Hospital (TASH) in Addis Ababa, Ethiopia, a national referral and teaching hospital. The study utilized a retrospective, hospital-based cross-sectional design, focusing on patients diagnosed with retinoblastoma from September 2018 to September 2021.

**Source and study participants**: The source population included all pediatric oncology patients evaluated at the Pediatric Oncology Unit of TASH during the study period. The study population specifically comprised patients under 14 diagnosed with retinoblastoma who underwent cross-sectional imaging and pathology evaluations. A convenience sampling method was used to include all patients meeting the inclusion criteria.

**Inclusion and exclusion criteria**: Inclusion criteria encompassed pediatric patients diagnosed with retinoblastoma who underwent CT or MRI, with corresponding pathology results. Patients with prior surgical history or trauma distorting the globe were excluded to avoid bias in imaging interpretation.

**Data collection procedure**: Patients were enrolled if they had both imaging and histopathology results available. Data were collected using structured questionnaires, which were pretested for clarity. The questionnaire extracted demographic data, clinical findings, and histopathology results. Imaging studies were performed using a Siemens Magnetom C 0.35T machine, 1.5T Philips MRI, and CT scans (124-slice Philips and 64-slice General Electric (GE). Two radiologists reviewed the images independently, with findings from bilateral cases reflecting the worst stage.

**Data analysis and interpretation**: Data were checked for completeness using Microsoft Excel and exported to SPSS (version 25) for analysis. Demographic characteristics, clinical symptoms, and imaging findings were summarized using tables. The IRSS was used to stage the disease based on cross-sectional imaging (Table 1), and the IIRC for intraocular disease classification (Table 2). Ethical clearance was obtained from the Department of Radiology's ethics committee, and patient identifiers were removed from the data.

**Table 1:** The international retinoblastoma staging system (IRSS) (10)

Stage	Clinical Description
0	Patient treated conservatively
I	Eye enucleated, completely resected histologically
II	Eye enucleated, microscopic residual tumor
III	Regional extension
a.	Overt orbital disease
b.	Preauricular or cervical lymph node extension
IV	Metastatic disease
a.	Hematogenous metastasis (without central nervous system involvement)
	1 Single lesion
	2 Multiple lesions
b.	Central nervous system extension (with or without any other site of regional or metastatic disease)
	1 pre-chiasmatic lesion,
	2 Central nervous system mass,
	3 Leptomeningeal and cerebrospinal fluid disease

**Table 2:** International Intraocular Retinoblastoma Classification (IIRC) (10)

Groups	International Intraocular Retinoblastoma Classification (IIRC)
Group A (very low risk)	All tumors are 3 mm or smaller, confined to the retina and at least 3 mm from the foveola and 1.5 mm from the optic nerve. No vitreous or subretinal seeding is allowed
Group B (low risk)	Eyes with no vitreous or subretinal seeding and discrete retinal tumor of any size or location. Retinal tumors may be of any size or location not in group A. The small cuff of subretinal fluid extending ≤5 mm from the base of the tumor is allowed
Group C (moderate risk)	Eyes with focal vitreous or subretinal seeding and discrete retinal tumors of any size and location. Any seeding must be local, fine, and limited to be theoretically treatable with a radioactive plaque. Up to one quadrant of subretinal fluid may be present
Group D (high risk)	Eyes with diffuse vitreous or subretinal seeding and/or massive, non-discrete endophytic or exophytic disease Eyes with more extensive seeding than Group C Massive and/ or diffuse intraocular disseminated disease including exophytic disease and >1 quadrant of retinal detachment. May consist of ‘greasy’ vitreous seeding or avascular masses. Subretinal seeding may be plaque-like
Group E (very high risk)	Eyes that have been destroyed anatomically or functionally with one or more of the following: Irreversible neovascular glaucoma, massive intraocular hemorrhage, aseptic orbital cellulitis, tumor anterior to anterior vitreous face, tumor touching the lens, diffuse infiltrating retinoblastoma and phthisis or pre-phthisis

## Results

The study included 83 participants, with 42 (50.6%) males and 41 (49.4%) females. Ages ranged from 1 to 12 years, with a mean of 3.4 ± 2 years. The most common presenting symptom was orbital swelling (proptosis) in 42 patients (50.6%), followed by leukocoria in 37 (44.6%). The mean duration of symptoms was approximately 5 ± 4 months. Regarding the affected eye, 37 (44.6%) exhibited symptoms in the left eye, 28 (33.7%) in the right eye, and 18 (21.7%) had bilateral involvement (Table 3).

**Table 3:** Primary clinical presentation and clinical staging based on IIRC of Retinoblastoma patients seen at TASH pediatric oncology department from Sept 2018- Sept 2021

Variables	Categories	Number	Percent
Clinical Presentation	Eye swelling	42	50.6
	Leukocoria	37	44.6
	Loss of Vision	1	1.2
	Eye Discharge	3	3.6
Involved Eye	Right Eye	28	33.7
	Left Eye	37	44.6
	Both Eyes	18	21.7
Clinical Staging	Group A	0	0
	Group B	0	0
	Group C	1	1.2
	Group D	19	22.9
	Group E	63	75.9

CT scans were performed for 27 (32.5%) patients, while 57 (68.7%) underwent MRI; one patient had both. Extraocular extension was found in 43 (51.8%) patients, with 20 (46.5%) limited to the intraconal space and 23 (53.5%) showing extraconal extension (Table 4). Calcification was identified in 23 (85.2%) of 27 patients on CT scans, with all cases exhibiting enhancement on post-contrast images (Table 5).

**Table 4:** Retinoblastoma local disease extent on CT and MRI of patients seen at TASH pediatric oncology department from Sept 2018- Sept 2021

Variables	Findings	Frequency (n=83) CT/MRI	Percent
Location	Intraocular	40	48.2
	Extraocular	43	51.8
		Intraconal=20	
		Extraconal=23	
Optic nerve invasion	Yes	65	78.3
	No	18	21.7
Intra cranial extension	Yes	24	28.9
		59	71.1

**Table 5:** Retinoblastoma imaging characteristics on CT and MRI of patients seen at TASH pediatric oncology department from Sept 2018- Sept 2021

Variables	Findings	CT scan (n=27)	Percent (%)	MRI(n=57)	Percent (%)
Lesion margin	Well defined	14	51.9	35	61.4
	Ill defined	13	48.1	22	38.6
Calcification	Yes	23	85.2		
	No	4	14.8		
Hemorrhage	Yes	7	26	10	17.5
	No	20	74	47	82.5
Enhancement	Present	27	100	57	100
	Absent	0		0	
Pattern of Enhancement	Homogenous	1	3.7	2	3.5
	Heterogeneous	26	96.3	55	96.5
T1 signal	Hypo intense	-		49	86
	Hyperintense	-		1	1.7
	Isointense	-		7	12.3
T2 signal	Hypo intense	-		37	65
	Hyperintense	-		8	14
	Isointense	-		12	21

Using the IRSS, disease staging revealed: 6 (7.2%) patients in Stage I, 12 (14.4%) in Stage II, 14 (16.9%) in Stage IIIA, 1 (1.2%) in Stage IIIB, 32 (38.5%) in Stage IVA, and 18 (21.7%) in Stage IVB (Table 6). Most patients (54, 65.9%) received a combination of chemotherapy and surgery, while 24 (29.3%) were treated solely with chemotherapy, 2 (2.4%) underwent surgery alone, and 1 (1.2%) received laser ablation.

**Table 6:** Staging of Retinoblastoma patients seen at TASH pediatric oncology department from Sept 2018- Sept 2021 using the International Retinoblastoma Staging System (IRSS)

IRSS Stages	Number (n)	Percent (%)
Stage I	6	7.2
Stage II	12	14.4
Stage IIIA	14	17
Stage IIIB	1 (0%)	1.2
Stage IVA	32	38.5
Stage IVB	18	21.7

## Discussion

A total of eighty-three pediatric patients from the age of 1 to 12 years took part in this study. The predominant clinical finding at the first presentation was proptosis. Notably, a significant majority of patients presented with an advanced stage, with almost 76% classified under the IIRC group E and 60.3% diagnosed with stage IV disease.

In the present study, a balanced gender distribution was observed among the patients, with a male-to-female ratio of 1.02:1. This finding aligns with similar studies conducted worldwide which had shown a lack of proof for any sex predilection for retinoblastoma (7, 8, 12). While certain studies in Asia, notably in India, have reported a higher incidence of retinoblastoma in males, such disparities may stem from gender-related healthcare access issues rather than inherent biological differences between the sexes (12). In the current study, the mean age at which children were diagnosed was approximately 39 months, signifying a considerable delay compared to studies conducted in more developed regions such as North America and Europe where the mean age of diagnosis was reported to be 12 months and 9 months, respectively(13, 14). However, the observed delay in diagnosis was consistent with findings from other studies conducted in various African countries, including Ethiopia (8, 15). This delay is attributable to a delayed recognition of symptoms by both parents and healthcare providers and lack of accessibility of the health services. Importantly, the delayed diagnosis has substantial implications for the prognosis of the affected children. Studies conducted in developing countries have demonstrated that a delay in diagnosis and initiation of treatment beyond 6 months can result in a marked increase in mortality, reaching up to 70% (6).

In our study, the predominant presenting sign of retinoblastoma was proptosis, with half of the children showing this sign at the time of presentation. This suggests an advanced stage of the disease at the time of diagnosis. Interestingly, this contrasts with clinical presentation in other studies conducted in North America, where leukocoria is identified as the most common presenting sign (16). However, our findings align with those of studies conducted in Cote-d'Ivoire and the Democratic Republic of the Congo (7).

In this study, 27 (32.5%) patients underwent head CT scan and 57 (68.7%) underwent brain MRI. MRI, with its superior soft tissue contrast and spatial resolution, holds an advantage, particularly in the follow-up of patients with established diagnoses (17). However, it is important to note that MR imaging may not be as specific as CT in detecting intraocular calcifications. To enhance diagnostic accuracy, additional sequences such as susceptibility sequences and in-phase imaging are often required, especially given that calcifications are a common finding in retinoblastoma patients (18).

The International Retinoblastoma Staging System (IRSS), established in 2006, categorizes the disease from stage 0 to stage IV, with stage 0 denoting purely intraocular disease and stage IV assigned to patients with metastasis (10). In our current study, utilizing the IRSS, the majority of patients presented with advanced disease, with 60.5% diagnosed at Stage IV. This aligns with findings from similar studies conducted in sub-Saharan Africa, suggesting that the prevalence of advanced extraocular disease at diagnosis may be attributed to delayed presentation and treatment initiation (7). To address this challenge, it is imperative to implement educational initiatives aimed at raising awareness about early signs of retinoblastoma. Additionally, instituting neonatal and pediatric ophthalmology examination programs, which involve testing for the red retinal reflex for leukocoria, has demonstrated benefits in the early detection of retinoblastoma, as supported by multiple studies (19). This study employed a retrospective design, which has several limitations, including the potential lack of comprehensive information in previously recorded data and challenges in controlling for confounding variables. Additionally, the CT and MRI findings and histopathology results were reported by different physicians, which may have introduced inter-reader variability.

Inconclusion that most patients presented with an advanced stage of retinoblastoma, with proptosis emerging as the most common presenting sign and symptom, and a substantial number of children diagnosed at Stage IV. There is a critical need to raise awareness about early signs of this disease like leukocoria and squint within the community and primary healthcare workers. It is also important integrating screening with the available pediatric, Maternal and Child Health (MCH) care or Expanded Program on Immunization (EPI) programs in Ethiopia. By fostering a greater understanding of the early signs and symptoms, we can encourage parents and caregivers to seek medical attention at earlier stages. This initiative-taking approach is crucial for improving the prognosis of affected children and underscores the importance of community-wide education initiatives on retinoblastoma. Furthermore, MRI and CT-scan machines are becoming available across various regional and zonal cities, so implementation of a standard early orbital imaging protocols and shortening referral system is advisable.

## Figures and Tables

**Figure 1:**
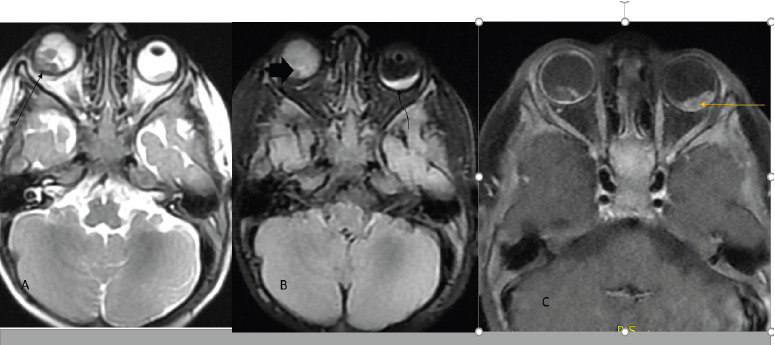
Ax T2W and FLAIR image showed right temporal quadrant intraocular mass extending to optic disc and suspicious focal extraocular extension and sign of vitreous seeding on right eye, Group D (line and fat arrows) and focal temporal quadrant lesion (curved arrow) on the left eye-Group B (A&B) and Ax T1FS+C showed heterogenous and nodular contrast enhancement (C)
